# Editorial Comment: Multiparametric Magnetic Resonance Imaging and Vesical Imaging-Reporting and Data System (VI-RADS) for Bladder Cancer Diagnosis and Staging: A Guide for Clinicians from the American College of Radiology VI-RADS Steering Committee

**DOI:** 10.1590/S1677-5538.IBJU.2026.9915

**Published:** 2026-07-08

**Authors:** Luciano A. Favorito

**Affiliations:** 1 Universidade do Estado do Rio de Janeiro Unidade de Pesquisa Urogenital Rio de Janeiro RJ Brasil Unidade de Pesquisa Urogenital - Universidade do Estado do Rio de Janeiro - Uerj, Rio de Janeiro, RJ, Brasil; 2 Hospital Federal da Lagoa Serviço de Urologia Rio de Janeiro RJ Brasil Serviço de Urologia, Hospital Federal da Lagoa, Rio de Janeiro, RJ, Brasil

Valeria Panebianco^1^, Alberto Briganti^2^, Jason A Efstathiou^3^, Samuel J Galgano^4^, Lyndon Luk^5^, Valdair F Muglia^6^, et al.

^1^ Department of Radiological Sciences, Oncology and Pathology, Sapienza University of Rome, Rome, Italy; ^2^ Unit of Urology/Division of Oncology, Urological Research Institute, IRCCS Ospedale San Raffaele, Vita-Salute San Raffaele University, Milan, Italy;^3^ Department of Radiation Oncology, Massachusetts General Hospital, Harvard Medical School, Boston, MA, USA; ^4^ Department of Radiology, University of Alabama at Birmingham Heersink School of Medicine, Birmingham, AL, USA; ^5^ Department of Radiology, Columbia University Irving Medical Center, New York, NY, USA; ^6^ Department of Medical Images, Radiation Therapy, Oncology and Hematology, Ribeirao Preto Medical School, University of Sao Paulo, Ribeirao Preto, Brazil

Eur Urol. 2026 Jun;89(6):487-499

DOI: 10.1016/j.eururo.2025.10.010 | ACCESS: 41176432

## COMMENT

Bladder cancer is one of the most common urologic cancers. This cancer has a high potential for aggression and about 1/3 of patients will be presented with muscle invasive disease, which is associated with a higher risk for metastatic disease. Treatment of the bladder cancer following transurethral resection will depend on the staging of the disease. Superficial tumors can be treated with intravesical therapy, whereas muscle-invasive tumors have conventional or robotic radical surgery as the treatment of choice (1-4).

The Vesical Imaging Reporting and Data System (VI-RADS), a scoring system emphasizing a standardized approach to multiparametric Magnetic Resonance Imaging (mpMRI) acquisition, has the purpose of to improve the diagnosis, staging and surveillance of patients with bladder cancer. mpMRI has been useful in the assessment of complete response after neoadjuvant chemotherapy and the response to immunotherapy (1).

In the paper of Panebianco (5), the authors show in a very interesting narrative review the importance of VI-RADS to bladder cancer diagnostic and therapeutic pathway. The authors show that mpMRI with VI-RADS improves the accuracy of preoperative staging, helps in avoiding repeat transurethral resection of bladder tumor in selected non muscular invasive bladder cancer cases, and supports decision-making in muscle invasive bladder cancer in MRI-based pathways. The authors concluded that mpMRI, VI-RADS and nacVI-RADS represent essential tools for enhancing risk stratification and personalizing bladder cancer management in clinical urology practice. I congratulate the authors on the importance of this paper.

## Data Availability

Data Not Provided
